# Non-destructive and accurate phenotypic detection method for okra seedlings under salt stress based on dual-view feature fusion and lightweight PointNet++

**DOI:** 10.3389/fpls.2026.1835129

**Published:** 2026-06-11

**Authors:** Siying Liu, Suhang Qian, Jingyi Tan, Jiamin Xu, Mei Lan, Ziyi Zhang, Haoqi Wang, Yuanyu Xia, Shihan Wang, Hongbin Wu, Xiuqing Fu

**Affiliations:** College of Engineering, Nanjing Agricultural University, Nanjing, China

**Keywords:** okra seedlings, phenotypic detection, point cloud segmentation, PointNet ++ model, salt stress

## Abstract

Soil salinization has become a critical factor limiting global agricultural production. Characterizing the growth and developmental responses of okra to salt stress and developing efficient and accurate salt-stress phenotyping techniques can provide an important methodological reference for okra cultivation in saline lands and future multi-cultivar salt-stress phenotyping studies. Traditional manual measurement of plant phenotypic parameters suffers from low efficiency and insufficient detection accuracy, making it difficult to achieve rapid and non-destructive analysis of plant phenotypic traits under salt stress. Therefore, this study proposes a computational phenotyping parameter extraction method based on the CSP-MSG Net model. Using dual-view feature fusion, we constructed a dedicated dataset. On the basis of PointNet++-MSG, the original MLP layers were replaced with C2F modules, and the SGE attention mechanism was integrated to enhance morphological feature extraction, thereby constructing a lightweight CSP-MSG Net architecture adapted to okra seedling point clouds for semantic segmentation of okra point clouds combined with DBSCAN clustering to complete instance segmentation, phenotypic parameters including plant height, stem diameter and canopy width were further calculated. This scheme enables high-throughput data acquisition, improves measurement accuracy, effectively reduces model parameters and computational overhead, and realizes lightweight operational performance. The results show that okra seedlings can still grow with increasing salt stress concentration, while the growth rates of the three measured traits are all inhibited, indicating that high-concentration salt stress impairs the growth activity of okra seedlings. To verify the calculation accuracy of the model, the phenotypic parameters predicted by the model were compared with manually measured values. The coefficients of determination for stem diameter, canopy width and plant height of okra seedlings reached 0.96, 0.99 and 0.99, respectively. These results strongly demonstrate the excellent reliability and effectiveness of the proposed method, providing methodological support for non-destructive and accurate phenotypic detection of okra seedlings under salt stress.

## Introduction

Soil salinization is a major abiotic limiting factor affecting plant growth and development and even regional distribution (CSIRO Plant Industry, GPO Box 1600, Canberra ACT 2601, Australia., 2002). At present, the problem of soil salinization is continuously intensifying, and multiple abiotic stresses induced by climate change have posed a serious threat to global food security (Suresh et al., 2026; Suresh et al., 2026). Meanwhile, it is predicted that the global food demand will double by 2050 to support nearly ten billion people, and multiple pressures bring enormous risks to ecologically vulnerable regions (Trigui et al., 2025).

Okra has high nutritional value and is rich in dietary fiber, mucilage, lysine, tryptophan and other essential amino acids, as well as linolenic acid. Moreover, okra is one of the most widely consumed vegetables worldwide and has the potential to adapt to diverse ecological environments. However, the intensification of salinization and climate change are seriously threatening the growth, yield and quality of okra (Ikram Ul et al., 2023).

The improvement and utilization of saline-alkali soil have long been a global challenge. Chinese researchers have carried out extensive and in-depth studies on crop salt stress response and phenotypic analysis. For okra, (Feibing et al., 2023) enhanced salt tolerance by regulating endogenous hormone metabolism, osmotic adjustment substances, photosynthetic pigments and ROS (Reactive Oxygen Species) metabolism (Wang et al., 2025). indicated that exogenous trehalose supplementation improves plant salt and drought tolerance by modulating osmotic regulators, protecting photosynthesis, maintaining ion homeostasis, and activating the ROS scavenging system. Current studies on the stress response of okra mainly focus on photosynthetic physiology, nutrient accumulation and fruit quality, while research on the effects of saline-alkali stress on its seed germination remains relatively limited. In this study, okra seeds were treated with NaCl solutions of different concentrations to analyze their germination characteristics and tolerance under saline-alkali conditions, aiming to provide methodological support for non-destructive phenotypic detection of okra under salt stress.

With the rapid development of smart agriculture and crop phenotyping technology, two-dimensional image-based crop phenotypic analysis has gradually become an important research direction in the field of smart agriculture. (Raja Ganapathy et al., 2026) combined the YOLO-nano model with thermal imaging technology and applied it to okra maturity grading and quality detection (Wang et al., 2024). acquired image data using ground spectrometers and unmanned aerial vehicles (UAV) equipped with digital cameras and conducted destructive sampling to determine LCC (Leaf Chlorophyll Content) and grain yield, providing a new method for estimating crop chlorophyll content and grain yield. (Bio-Sensing and Instrumentation Laboratory, College of Engineering, University of Georgia, Athens, Georgia, United States of America. et al., 2019) extracted plant height, canopy coverage and flower number from UAV multispectral two-dimensional images to establish the correlation between phenotype and yield.

With the advancement of 3D reconstruction and sensor technologies, point cloud-based plant structural modeling has become an important approach for automatic extraction of crop phenotypic parameters (Zhou et al., 2026). To address the challenges brought by the complexity of point cloud data, researchers have gradually introduced deep learning methods into structural recognition and semantic segmentation of plant 3D point clouds (Guo et al., 2020). (Omia et al., 2026) systematically reviewed the research progress of point cloud-based three-dimensional phenotyping of field crops, compared the performance of various existing sensor technologies in controlled field environments, deeply analyzed the technical challenges in controlled and field scenarios, and put forward optimization suggestions focusing on illumination fluctuation, crop occlusion, wind disturbance, uneven point cloud density and noise interference in field conditions (Liu T. et al., 2026). realized high-precision organ-level point cloud segmentation of individual plants, which can accurately distinguish key plant organs such as leaves, stems and fruits. It effectively solves common field problems including uneven point cloud density, noise interference and organ occlusion, providing a reliable segmentation basis for subsequent phenotypic analysis. At present, the PointNet++ network has become a mainstream architecture for industrial point cloud analysis (Sun et al., 2026).

In practical applications, despite the continuous development of sensor technology, the acquired point cloud data are still prone to uneven density, noise interference and target occlusion, which increase the difficulty of 3D modeling and phenotypic parameter extraction. Therefore, it is necessary to introduce point cloud processing methods with stronger structural modeling and scene perception capabilities to improve the accuracy of parameter extraction. Accordingly, this study carried out lightweight improvement on the PointNet++ network to more efficiently extract and quantitatively characterize the three-dimensional morphological features of okra seedlings. Meanwhile, based on PointNet++-MSG, the CSP-MSG Net architecture suitable for okra phenotypic analysis was constructed. The C2F module (Qin et al., 2025) generally adopts progressive inference to quickly locate key seedling regions, reduce redundant computation, and accurately capture fine-grained features. It effectively reduces computational consumption and improves the efficiency and robustness of three-dimensional phenotypic analysis, making it more suitable for lightweight networks compared with other modules. By replacing the MLP layers with C2F layers, integrating the SGE attention mechanism module (Chen et al., 2025), and introducing the DBSCAN clustering algorithm, the proposed model achieves stronger feature extraction capability and higher computational efficiency while better retaining detailed information. By analyzing the phenotypic traits of individual seedlings under different attention mechanisms (Wang et al., 2026), the high efficiency and accuracy of the model were verified. Furthermore, experiments on seedling growth under different salt stresses were conducted. Based on seedling point cloud data and the CSP-MSG Net model, the phenotypic traits and growth rules of okra seedlings under different salt concentrations were analyzed.

## 2 Materials and methods

### 2.1 Introduction of experimental equipment

**Figure 1:**
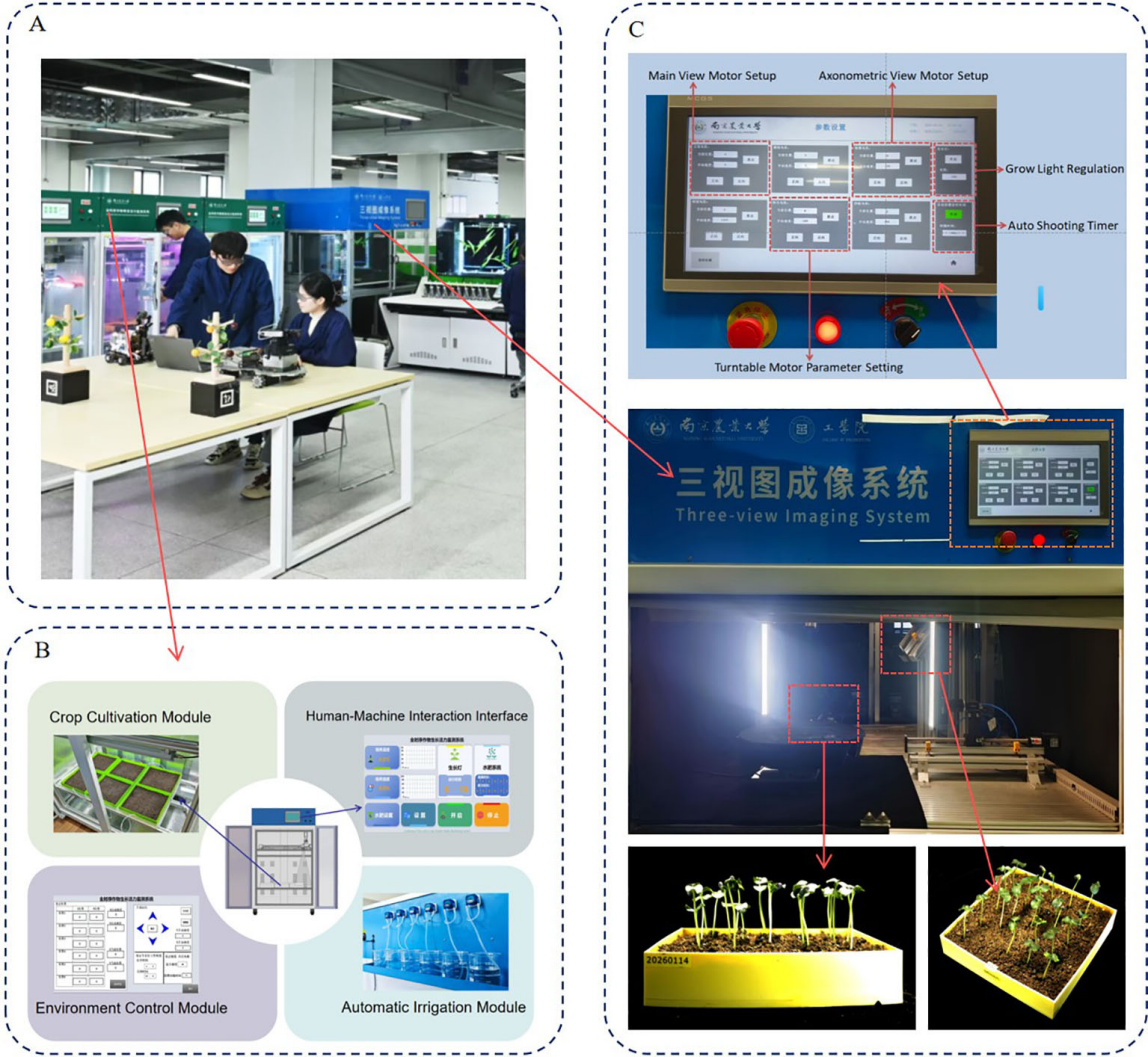
**(A)** Experimental scene; **(B)** full-time crop growth vitality monitoring system; **(C)** Three-view imaging system.

The seed cultivation equipment adopts the full-time crop growth vitality monitoring system, as shown in **Figure 1B**. In this experiment, the environmental control module, automatic irrigation module and crop cultivation module of the system were mainly used to realize the accurate simulation of crop growth conditions in an indoor environment. The environmental control module monitors real-time data such as temperature, humidity and CO_2_ concentration inside the chamber through built-in temperature and humidity transmitters, ensuring that seeds are maintained in a suitable growth environment. The automatic irrigation module delivers the solution in beakers to the bottom of seedling trays via six peristaltic pumps installed at the rear of the chamber, realizing timed and quantitative automatic irrigation. The crop cultivation module is supported by a metal frame and equipped with LED plant growth lamps at the top to provide controllable light conditions for crops. Six square seedling trays are used for seed cultivation. The human–computer interaction interface displays monitoring data, equipment status and operation buttons in separate zones, enabling rapid acquisition of core information and manual regulation of environmental parameters such as temperature, humidity and light intensity.

**Figure 2:**
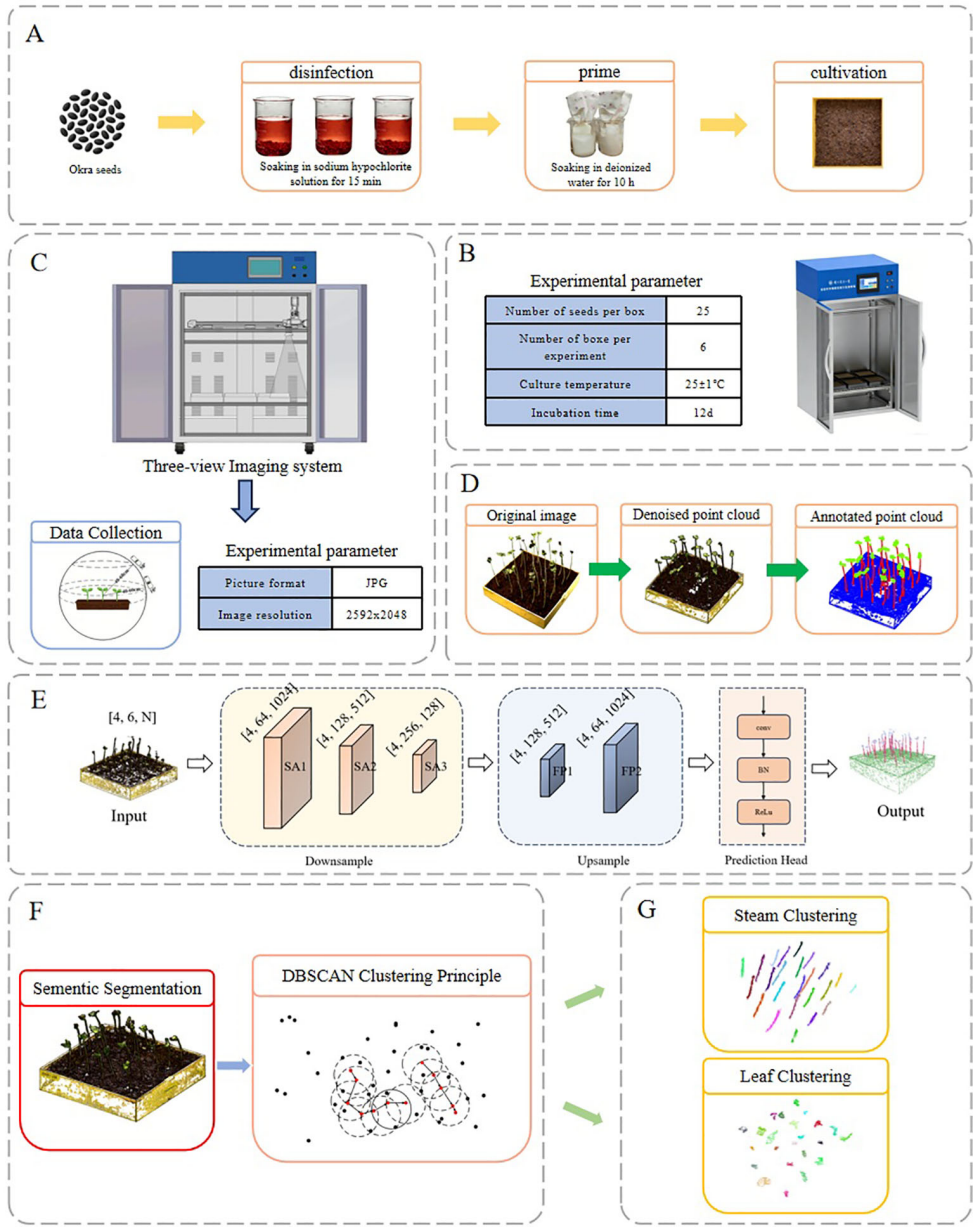
**(A)** Seed pretreatment process; **(B)** seed cultivation; **(C)** data acquisition; **(D)** data processing and annotation; **(E)** overview of the CSP-MSG net model; **(F)** principle of DBSCAN instance segmentation; **(G)** visualization of instance segmentation results.

The data acquisition equipment adopts a three-view imaging system, as shown in **Figure 2C**. It is mainly composed of a high-precision electric rotating platform, industrial cameras, fixed brackets and a main control computer. The system is equipped with Hikvision MV-CA050-20GC industrial vision cameras, including one front-view, one oblique side-view and one top-view camera. The cameras can capture extremely subtle image details, making them suitable for high-precision measurement and three-dimensional reconstruction. Potted okra seedlings were placed on the electric rotating platform to achieve precise 360°continuous rotation. Meanwhile, sequential dual-view imaging was performed to acquire high-precision image data for three-dimensional morphological analysis of okra. The captured images are stably transmitted through the GigE interface, laying a foundation for subsequent image processing.

### 2.2 Dataset construction

#### 2.2.1 Seed cultivation

Okra seeds of Lingyun No. 2 were selected in this experiment. The seed pretreatment process is shown in **Figure 2A**. Firstly, plump and disease-free okra seeds were disinfected. Subsequently, the seeds were neatly placed on filter paper, wrapped with filter paper rolls, and soaked in deionized water for constant-temperature priming for 10 hours. The filter paper roll wrapping allowed the seeds to be exposed to air while obtaining sufficient water supply. Afterwards, the seeds were evenly arranged in a 5×5 layout in square seedling trays with a side length of 23 cm and placed into the full-time crop growth vitality monitoring system for cultivation. The experimental parameters are presented in **Figure 2B**.

#### 2.2.2 Data acquisition and processing

Data acquisition was initiated when okra seedlings grew approximately 3–4 cm above the soil surface. The planar morphological information provided by the front view and the vertical depth information provided by the oblique side view is highly complementary, which can provide sufficient geometric constraints for point cloud reconstruction and completely restore the three-dimensional morphology of okra seedlings. Adding a top view introduces substantial information redundancy, contributes little to the improvement of reconstruction accuracy, and instead increases the complexity of data processing. Therefore, considering both accuracy and efficiency, only the front view and oblique side view were adopted for three-dimensional reconstruction in this study. As shown in **Figure 2C**, potted seedlings were placed into the three-view imaging system to capture video images from the front view and oblique side view respectively. To ensure temporal and spatial consistency of data from different perspectives, a turntable reset step-by-step acquisition scheme was adopted to achieve equivalent synchronization. After the front-view shooting was completed, the platform automatically reset to the initial angle, and the oblique side-view camera repeated the same rotational acquisition procedure with consistent parameter settings throughout the whole process. Each video lasted about 2 minutes, and shooting was performed at an interval of 24 hours. After manually eliminating blurred, severely occluded and abnormally captured samples, a total of 108 high-quality video groups were finally screened from 120 groups (each group contained videos of one potted okra seedling from two perspectives) for subsequent three-dimensional reconstruction and dataset construction.

For the screened valid video dataset, frame extraction was first conducted for each video: one frame was extracted every 3°for the oblique side-view video and every 6°for the front-view video, with approximately 180 images extracted from each video. Subsequently, the COLMAP three-dimensional reconstruction software was used to reconstruct the three-dimensional point cloud of each potted okra seedling by combining front-view and oblique side-view images, ensuring the integrity and accuracy of the reconstructed point clouds. Ultimately, 108 three-dimensional point cloud files in PLY format were obtained. Finally, CloudCompare software was used to process all point cloud files to remove redundant noise points and perform manual point cloud annotation. In the annotation scheme, label 0 represents the seedling tray, label 1 represents okra seedling leaves, and label 2 represents okra seedling stems. After annotation, the labeled point cloud data were exported in a unified TXT format to construct a standardized three-dimensional point cloud dataset for model training and validation. This provides high-quality annotated samples for organ-level segmentation, automatic phenotypic parameter extraction and accuracy verification of okra seedlings based on deep learning models such as PointNet++. The specific workflow is illustrated in **Figure 2D**. **Figure 2E** shows the structural framework of the proposed CSP-MSG Net model for subsequent semantic segmentation tasks. **Figures 2F**, **G** present the instance segmentation results of okra seedling point clouds using the DBSCAN algorithm.

To verify the accuracy of phenotypic parameters including plant height, stem diameter and canopy width measured by the model in the later stage, manual measurement was performed for each seedling immediately after daily data collection, and the measured data were recorded for comparison with model outputs. For plant height, the vertical distance from the root base to the top growth point of the seedling is measured with a ruler. For stem diameter, a vernier caliper is used to measure the thickest part of the stem below the cotyledon, and the average value of three repeated measurements is taken to reduce errors. For canopy width, the maximum horizontal span of the seedling canopy is measured with a straight ruler at its widest extension. By synchronously collecting manual measured data and model output data every day, a comparative dataset was established, providing reliable measured support for subsequent model accuracy evaluation, error analysis and model optimization.

#### 2.2.3 Data augmentation

To improve the robustness and generalization ability of the model in okra seedling point cloud segmentation, data augmentation was performed on the annotated original seedling point clouds. The augmentation strategies adopted in this study mainly include rotation perturbation, local deformation perturbation, background noise injection, voxel random dropping, and adjacency graph reconnection. Rotation perturbation simulates placement deviations during sampling on the rotating platform by applying a random rotation of ±15° around the vertical axis. Local deformation perturbation imposes controllable and slight local deformation on seedling point clouds to prevent model overfitting and simulate differences in seedling posture and slight morphological variations under natural growth conditions. Background noise injection randomly adds 5%–15% soil clutter points to simulate interference caused by leaf reflection and background soil noise. Voxel random dropping discards 12%–22% voxel units from voxelized point clouds, simulating leaf occlusion, light reflection, and blind areas in three-dimensional reconstruction. Adjacency graph reconnection randomly disconnects and reconstructs partial original topological connections, reducing the model’s dependence on fixed local connections and enhancing its attention to the overall morphological structure of crops. These strategies effectively avoid model overfitting to ideal clean point cloud data, enrich training samples, and promote generalization performance. In this study, three biological replicates were conducted, with six salt concentration treatments set for each replicate. Okra seedlings of each concentration were cultivated in an independent tray and continuously photographed for six consecutive days, yielding a total of 18 independent trays and 108 observation videos. Trays were randomly divided at the tray level: all video sequences from 14 trays were assigned to the training set, and the remaining 4 trays to the test set, so as to prevent data leakage.

### 2.3 Proposal of the CSP-MSG net model

**Figure 3:**
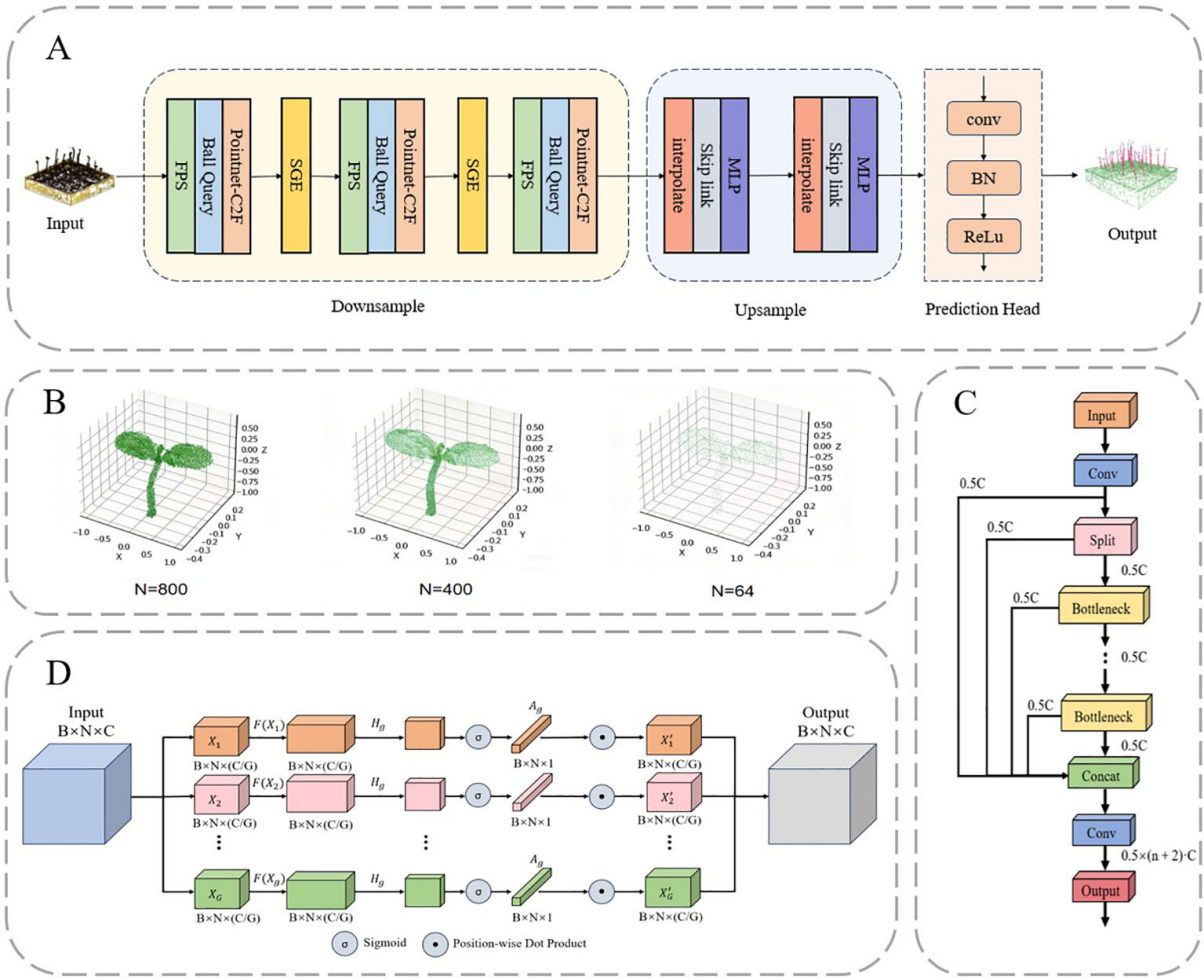
**(A)** Basic architecture of the CSP-MSG Net model; **(B)** Principle of FPS farthest point sampling; **(C)** Architecture of the C2F module; **(D)** Architecture of the SGE attention module.

In the feature extraction process of the original PointNet network (Huang Y. et al., 2025), independent feature calculation and extraction are first performed on each point of the 3D point cloud, followed by global max pooling to aggregate all point features. Such a mechanism is difficult to effectively capture fine local features of key components such as leaves and stems in okra seedling point clouds. As an improved version of PointNet, PointNet++ (Liu W. et al., 2026) constructs a hierarchical feature extraction structure and adopts a multi-scale local aggregation mechanism. It shows higher adaptability in fine-grained feature extraction of okra seedling point clouds and can better describe local morphological details. Nevertheless, PointNet++ still has obvious limitations. During local feature learning, it mainly extracts coordinate differences between points while ignoring rich local geometric semantic information inherent in okra point clouds, such as curvature, normal vectors and neighborhood distribution. Moreover, its feature extraction relies excessively on a fixed local neighborhood range, lacking the ability to model long-range correlations among points. This makes it difficult to capture structural dependencies within the overall okra seedling morphology and further impairs the learning of complete semantic features from point clouds. PointNet++-MSG is a multi-scale grouping variant of PointNet++ designed for semantic segmentation tasks. However, it still suffers from insufficient differentiated weighting of feature channels and a single feature transformation path in MLP layers. To better meet the requirement of fine feature extraction for okra seedling point clouds, this study conducts modular improvement and scenario adaptation based on PointNet++-MSG and constructs the CSP-MSG Net architecture. Its working principle is shown in **Figure 3A**.

#### 2.3.1 Replacing MLP layers with C2F module

PointNet++-MSG adopts a hierarchical sampling strategy, which is mainly divided into the downsampling process (SA module) and the upsampling process (FP module). In the CSP-MSG Net model, three stacked SA modules and two stacked FP modules are used to complete the transformation from dense okra point clouds to sparse high-order features and restore the number of original point clouds. The MLP layer serves as the core unit for feature learning in the SA module. However, the original MLP in the SA module designs an independent deep network for each scale and lacks feature interaction. By contrast, adopting a lighter and more efficient CSP structure to extract local point cloud features can effectively improve the multi-scale feature utilization of PointNet++, while reducing network parameters and computational complexity via the lightweight Bottleneck structure. Therefore, this study adapts the C2F module to okra point cloud processing scenarios to replace the independent MLP feature extraction layers within each scale branch of the SA module in PointNet++-MSG. This improves the computational efficiency and robustness of the model and constructs the PointNet++-MSG-C2F model.

As the core part of the PointNet++-MSG-C2F model, the detailed downsampling procedure is described as follows:

##### 2.3.1.1 Farthest point sampling

Farthest Point Sampling (FPS) (Duan et al., 2024) is a uniform point cloud sampling algorithm. It iteratively selects the point farthest from the already sampled set, enabling sampled points to be uniformly distributed in space and maximally cover the geometric structure of the original point cloud. It achieves uniform point cloud downsampling and provides a centrally distributed point set with uniform spatial layout for subsequent hierarchical feature extraction. An example of FPS sampling is shown in **Figure 3B**. Let the input point cloud be $\text{P}=\{{\text{p}}_{1},{\text{p}}_{2},\ldots ,{\text{p}}_{\text{N}}\}\subset R^{3}$, and the sampled point set be ${\text{S}}_{\text{k}}=\{{\text{s}}_{1},{\text{s}}_{2},\ldots ,{\text{s}}_{\text{k}}\}\subset \text{P}$. The (k+1)-th sampling point of FPS is formulated as **Equation 1**:

(1) $${\text{s}}_{\text{k}+1}={\text{argmax}}_{{\text{p}}_{\text{i}}\in \text{P}/{\text{S}}_{\text{k}}}(\min_{\text{s}\in {\text{S}}_{\text{k}}}∥{\text{p}}_{\text{i}}-\text{s}{∥}_{2})$$

where ∥·∥ represents the Euclidean distance, and P/S_k_ denotes the unsampled point set.

##### 2.3.1.2 Multi-scale grouping

Different from Single-Scale Grouping (SSG) (Cong et al., 2026), after FPS sampling in the SA module, MSG sets multiple spherical query neighborhoods with different radii for the same sampling center in each SA module. For a given center point, the radius r is the core parameter of spherical query, defining all points within a sphere centered at this point with radius r, i.e., the local neighborhood range of the center point. Different radii correspond to local feature extraction at different scales: a small radius captures fine-grained local geometric details, while a large radius acquires macroscopic structural information. Features of each scale are then concatenated and fused, enabling the network to perceive geometric information across multiple scales simultaneously. To unify feature dimensions, each neighborhood is fixed to K points by limiting the neighborhood size or zero-padding, forming a feature dimension of $R^{\text{B}\times \text{C}\times \text{M}\times \text{K}}$. Here, B denotes batch size, C denotes input feature channel number, M denotes the number of sampled center points, and K denotes the fixed point number per neighborhood.

##### 2.3.1.3 C2F cross-scale feature encoding

The structure of the C2F module, which replaces the original MLP layers in PointNet++-MSG within the CSP-MSG Net model, is illustrated in **Figure 3C**. For the input point cloud, the coordinates and original features of neighborhood points are first concatenated, and a 1×1 convolution is applied to adjust the channel number to 2C(C= $\left\lfloor {\text{C}}_{\text{out}}\cdot \text{e}\right\rfloor$, he channel expansion ratio set to 0.5 in this study) for subsequent feature splitting,which is calculated via **Equation 2**:

(2) $${\text{F}}_{\text{conv}}={\text{Conv}}_{1\times 1}(\text{Concat}({\text{F}}_{\text{in}},\text{X}))\in R^{\text{N}\times 2\text{C}}$$

where ${\text{F}}_{\text{in}}\in R^{\text{N}\times {\text{C}}_{\text{in}}}$ denotes the input feature matrix, N is the number of point clouds, C_in_ is the input channel number, and Concat(·) represents channel-wise concatenation.

A divide-and-conquer strategy is then adopted. The adjusted features are equally divided along the channel dimension into a direct branch F_direct_ and a calculation branch F_calc_. Features in the direct branch skip deep transformation, while features in the calculation branch undergo deep feature learning by stacking n Bottleneck blocks. The output of each Bottleneck is preserved as an independent branch. The Bottleneck is a lightweight residual bottleneck unit specially used for internal feature extraction of the C2F module. Aiming at okra point cloud recognition and segmentation tasks, the Bottleneck block is optimized in this study. The original three-layer convolution structure is replaced by a lightweight structure composed of two 1×1 convolutions, which satisfies task requirements while greatly reducing computational consumption. Finally, features from the direct branch and all calculation branches are concatenated along the channel dimension and mapped to the target output dimension via a 1×1 convolution,as mathematically expressed in **Equation 3**:

(3) $${\text{F}}_{\text{out}}={\text{Conv}}_{1\times 1}(\text{Concat}({\text{F}}_{\text{direct}},{\text{F}}_{1},{\text{F}}_{2},\ldots {\text{F}}_{\text{n}}))\in R^{\text{N}\times {\text{C}}_{\text{out}}}$$

where Concat denotes the concatenation operation and C_out_ is the target output channel number; n=1 is adopted in this study.

##### 2.3.1.4 Feature aggregation

The C2F output features of the three scales are concatenated along the channel dimension, followed by Max Pooling neighborhood aggregation to obtain high-order semantic features of each center point. This achieves the downsampling objective of reducing point number while enhancing feature representation.

#### 2.3.2 Introduction of SGE attention mechanism module

The Spatial Group-wise Enhance (SGE) module (Hu et al., 2026) adopts a semantic group-based attention mechanism, in which the attention factor depends only on the similarity calculation of global and local feature descriptors within each group. Based on the PointNet++-MSG-C2F model, the SGE attention module is inserted after the output of the feature extraction subnetwork in each SA module and before the input to the next SA module. The detailed structure is shown in **Figure 3D**.

A key bottleneck of semantic segmentation tasks is that the model tends to focus on irrelevant regions while ignoring critical semantic regions of targets, resulting in low segmentation accuracy for object details. The prominent advantage of SGE lies in that, at each stage of hierarchical feature extraction, the network can perform grouped enhancement on feature channels at the current scale. Channels related to the key morphological features of okra seedlings are assigned higher response weights, whereas channels associated with noise and redundant information are appropriately suppressed, thereby forming the final CSP-MSG Net model. The specific processing procedure is formulated in **Equation 4** as follows.

Given the input feature $\text{X}\in R^{\text{B}\times \text{N}\times \text{C}}$, SGE first equally divides the channel dimension into G groups:

(4) $$\text{X}=[{\text{X}}_{1},{\text{X}}_{2},\ldots ,{\text{X}}_{\text{G}}],{\text{X}}_{\text{g}}\in R^{\text{B}\times \text{N}\times \text{C}/\text{G}}$$

where X_g_ denotes the feature map of the g-th group; B is the batch size, N is the number of points, and C is the number of feature channels. For each group X_g_, a lightweight point cloud spatial encoding function F is adopted. SGE performs spatial feature encoding on each group feature to aggregate contextual information, as defined in **Equation 5**:

(5) $${\text{F}}_{\text{g}}=ℱ({\text{X}}_{\text{g}})\in R^{\text{B}\times \text{N}\times \text{C}/\text{G}}$$

where F denotes the lightweight spatial feature encoding function. Subsequently, a spatial attention map corresponding to each group is generated via convolution and activation function, whose mathematical expression is given in **Equation 6**:

(6) $${\text{A}}_{\text{g}}=\text{σ}(H_{\text{g}}({\text{F}}_{\text{g}}))\in R^{\text{B}\times \text{N}\times 1}$$

where $H_{\text{g}}$ represents the lightweight convolution layer, and σ denotes the Sigmoid activation function. The attention map is element-wisely multiplied with the corresponding group feature to obtain the enhanced feature ${\text{X}}_{\text{g}}^{'}$, as calculated in **Equation 7**:

(7) $${\text{X}}_{\text{g}}^{'}={\text{X}}_{\text{g}}⊙{\text{A}}_{\text{g}}$$

Finally, the enhanced features of all groups are concatenated to yield the final output feature as expressed in **Equation 8**:

(8) $$\text{Y}=\text{Concat}([{\text{X}}_{1}^{'},{\text{X}}_{2}^{'},\ldots ,{\text{X}}_{\text{G}}^{'}])$$

Where *Concat* denotes the channel concatenation operation.

### 2.4 DBSCAN clustering segmentation

In this study, the DBSCAN clustering algorithm (Garah et al., 2026) was adopted to realize clustering segmentation of leaves and stems of whole-potted okra seedlings, laying a foundation for the subsequent acquisition of phenotypic traits of okra seedlings.

DBSCAN is a density-based clustering algorithm, and its principle is illustrated in **Figure 2F**. It does not require a predefined number of clusters but automatically discovers clusters of arbitrary shapes according to the density distribution of data points, and can effectively identify noise points with strong robustness. The neighborhood radius eps and the minimum number of points minPts required to form a cluster are the core parameters of DBSCAN, which largely determine the algorithm performance. In this experiment, eps was set to 3 and minPts was set to 10. Specifically, taking any sample point in the dataset as the center, a sphere with a radius of 3 is constructed. If the sphere contains no fewer than 10 sample points (including the center point itself), the center point is defined as a core point. DBSCAN traverses each point in the dataset and aggregates all points within the neighborhood of core points (including core points) into one cluster. Points that are neither core points nor within the neighborhood of any core point are regarded as noise points. Visualization performance is an important indicator to verify the effectiveness of the clustering algorithm, and the visualization results of instance segmentation for okra seedlings are shown in **Figure 2G**.

### 2.5 Calculation of phenotypic parameters of okra seedlings

#### 2.5.1 Scale recovery

**Figure 4:**
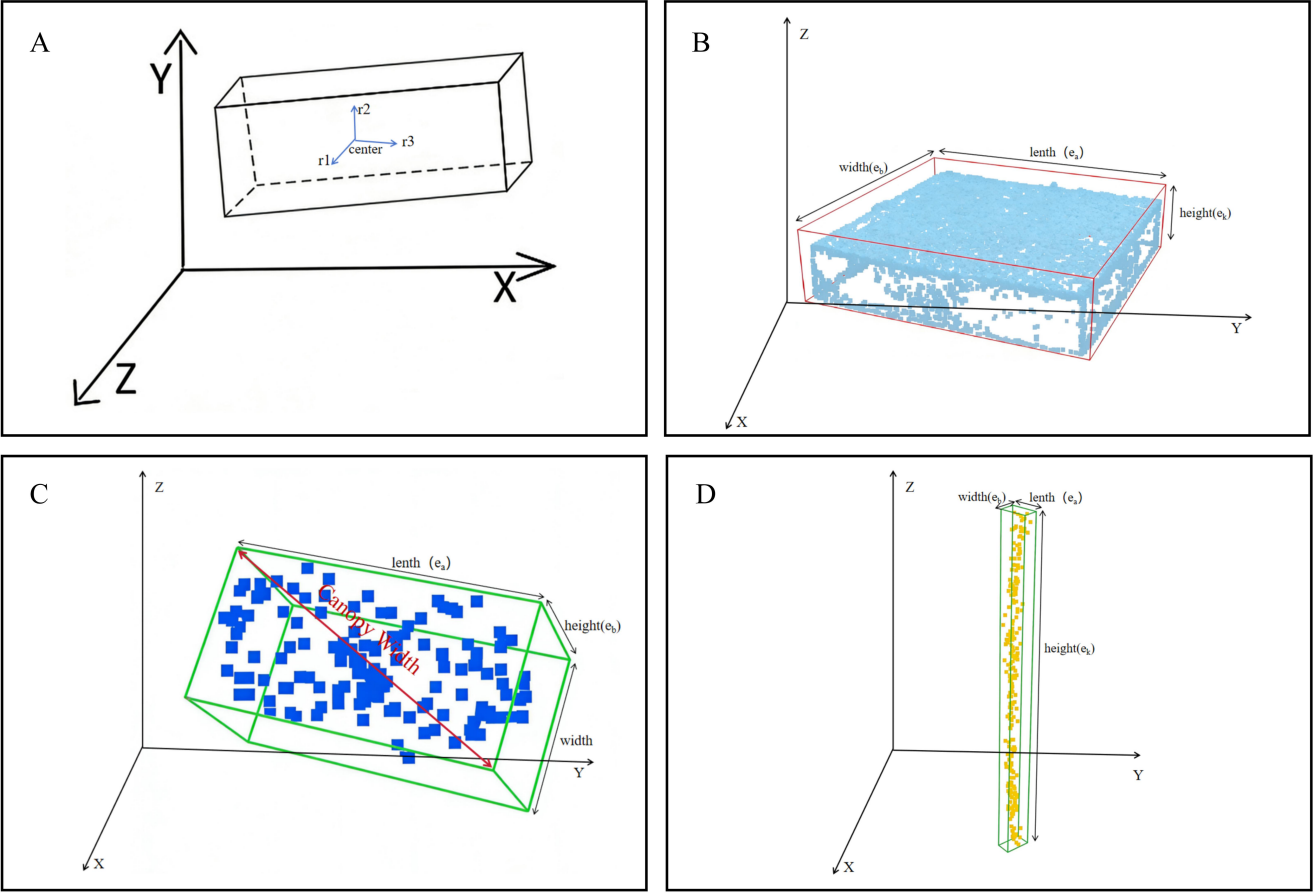
**(A)** OBB bounding box; **(B)** scale recovery method using OBB-enclosed seedling tray; **(C)** calculation method of canopy width; **(D)** OBB bounding of stem point cloud.

The 3D point clouds reconstructed by CloudCompare software (Sanchez Aparicio et al., 2025) only retain relative geometric structures without real physical scale information. Consequently, true phenotypic parameters such as plant height and stem diameter of okra seedlings cannot be directly acquired, which imposes certain impacts on subsequent experimental evaluation. To address this issue, an oriented bounding box (OBB) (Tomiło, 2026) method based on Principal Component Analysis (PCA) (Pandhi and Kumar, 2025) was introduced. Taking advantage of its superior capability in 3D geometric feature extraction and combined with a scale calibration strategy, the reconstructed unscaled point clouds were further processed. The real seedling dimensions were inversely derived according to the calculated scale calibration coefficient (Zhu et al., 2025). The core principle of the OBB algorithm is to tightly enclose an object with a rotated cuboid. Essentially, OBB is a rectangular bounding box with three dimensions: length, width, and height, as shown in **Figure 4A**. It can freely rotate and scale around the origin of the three-dimensional coordinate system according to the actual geometric morphology of the target object, enabling the six faces of the cuboid to fit the object surface as closely as possible. Its length, width, and height correspond respectively to the scales of the first, second, and third principal axes of the bounding box. The mathematical expression of OBB is defined in **Equation 9** as:

(9) OBB=center+R·diag(hx,hy,hz)

where center denotes the center point of the bounding box; R represents the rotation matrix determined by the eigenvectors of the covariance matrix of the object point set; hi refers to the half-length along the i-th axis, which is calculated from the extreme values of projected vertices.

Point cloud data can obtain the rotation matrix R through eigenvalue decomposition of the covariance matrix, so as to align the point cloud with the principal axes of the OBB bounding box. The covariance matrix is an important mathematical tool for characterizing the spatial distribution of multidimensional data, and its elements reflect the linear correlation between different data dimensions. For a 3D point cloud dataset, the covariance matrix C is calculated via **Equation 10** as follows:

(10) $$\text{C}=\frac{1}{n}\sum_{\text{i}=1}^{\text{n}}({\text{p}}_{\text{i}}-\text{μ}){\left({\text{p}}_{\text{i}}-\text{μ}\right)}^{\text{T}}$$

where μ is the mean of the point set, and p_i_ denotes the coordinate of each vertex. In the OBB algorithm, the eigenvectors of the covariance matrix define the principal directions of the bounding box, while the eigenvalues correspond to the length of each axis.

In the experiment, a seedling tray with known physical dimensions was used as the calibration object, with a length of 23 cm, a width of 23 cm, and a height of 5 cm. The 3D point cloud of the seedling tray was then enclosed by the OBB, as shown in **Figure 4B**. Taking the seedling tray of one potted seedling as an example, the main parameters of the OBB are listed in **Table 1**. The scale calibration coefficient k was calculated from the real physical dimensions of the seedling tray and its virtual point-cloud dimensions, namely the length, width and height of the OBB. Afterwards, the overall and local point clouds of okra seedlings were separately enclosed by OBBs, and the real phenotypic parameters of seedlings were obtained by multiplying the virtual dimensions by the coefficient k.

**Table 1:** Main data composition of the OBB bounding box.

OBB bounding box parameters	Specific values
OBB Length (First Principal Axis)	1.7621854570591762
OBB Width (Second Principal Axis)	1.7705448579012777
OBB Height (Third Principal Axis)	0.346495879732133
OBB Center	[0.0021641, 0.18514387, 0.15247449]
OBB Rotation Matrix (Column Vectors as Principal Axis Directions)	[-0.46051815, -0.88754841, 0.01344791] [0.63886089, -0.32088863, 0.6992047] [-0.61626277, 0.33058782, 0.714795]

#### 2.5.2 Calculation method of plant height

The plant height of okra seedling is defined as the vertical distance from the ground to the seedling apex (Bazrafkan et al., 2025). To calculate plant height, the highest and lowest points of the seedling are first determined, and the Euclidean distance between the two points is regarded as the plant height. Considering the natural inclination of okra seedlings during growth, the OBB bounding box method was adopted in this study to improve measurement accuracy. Essentially, this method extracts the maximum vertical length of the plant by utilizing the orientation and dimension of the OBB. The specific calculation procedure is as follows: For the point cloud of a single okra seedling, the oriented bounding box (OBB) is firstly calculated. Let the rotation matrix of OBB be R = [r_1_, r_2_, r_3_] and the corresponding axis length be e = [e_1_, e_2_, e_3_]. The included angle between each principal axis and the Z-axis of the world coordinate system is calculated as: θ = arcos(r_i_·[0,0,1]^T^),the principal axis with the minimum angle is selected as the vertical principal axis k. Finally, the plant height H is defined as the bounding box length corresponding to this principal axis: H = e_k_. Geometric derivation indicates that when the inclination angle is 10°, the theoretical deviation between the OBB principal axis length and the true vertical plant height is only 1.5%. Such deviation is far smaller than the actual variation range of plant height (approximately 15%–30%) under different salt concentrations, and thus barely affects the statistical analysis of salt stress effects.

#### 2.5.3 Calculation method of canopy width

Canopy width is one of the core indicators for evaluating seedling growth status (Zhou et al., 2025), which directly reflects the growth vigor and health condition of okra seedlings (Tian et al., 2025).For canopy width measurement based on OBB, the point cloud of the okra seedling leaf region is enclosed by an oriented bounding box, as shown in **Figure 4C**. The lengths of the two principal axes of the OBB on the horizontal plane are then extracted. First, the vertical principal axis k is identified and excluded using the same method described for plant height calculation. The remaining two principal axes lie in the horizontal plane, where the longer axis corresponds to the canopy major axis (e_a_) and the shorter axis corresponds to the canopy minor axis (e_b_).

To improve calculation accuracy, the canopy width is defined as the diagonal length of the bounding box in the horizontal plane, calculated via **Equation 11** as:

(11) $$\text{Canopy Width}=\sqrt{{\left({\text{e}}_{\text{a}}\right)}^{2}+{\left({\text{e}}_{\text{b}}\right)}^{2}}$$

Morphological analysis based on the dataset in this study shows that the ratio of the OBB major axis to minor axis of okra seedling canopy ranges approximately from 1.05 to 1.10. Geometric derivation indicates that the difference between the diagonal length and the maximum lateral span is only 1.2%–4.5%. This error is far smaller than the canopy variation caused by salt stress treatments and therefore has negligible influence on subsequent analytical results.

#### 2.5.4 Calculation method of stem diameter

Stem diameter (Delapierre et al., 2024) refers to the stem diameter at the base or middle part of seedling stems, which is a key morphological indicator reflecting seedling robustness and lodging resistance. A larger stem diameter indicates stronger seedling growth vigor and better health status. In this study, the OBB bounding box method was adopted to calculate stem diameter. Firstly, the stem of each seedling was separated from the whole plant, which had already been completed through instance segmentation using the DBSCAN clustering algorithm (Yu et al., 2025). Different from the OBB calculation for canopy width, only the point cloud of the stem region was enclosed for stem diameter measurement, as shown in **Figure 4D**. The corresponding rotation matrix *R* and principal axis lengths *e* were obtained by fitting the OBB. The vertical principal axis was determined and excluded by calculating the angle between each principal axis and the vertical direction. The stem diameter was defined as the average length of the remaining two horizontal principal axes, and the formula is expressed in **Equation 12** as:

(12) $$D=\frac{e_{a}+e_{b}}{2}$$

To verify the robustness of the stem diameter extraction method against segmentation noise, Gaussian noise with a standard deviation of 1%–5% of the true stem diameter was added to the stem contour to simulate the uncertainty of segmentation boundaries. The results show that even at a noise level of 5%, the calculation error of stem diameter remains less than 1%, demonstrating that the proposed method possesses favorable capability to suppress segmentation noise.

### 2.6 Training environment and model evaluation metrics

#### 2.6.1 Training environment

The operating system used in this experiment was Windows 11. The hardware configuration included an NVIDIA GeForce RTX 4050 Laptop GPU and an Intel Core i7-13620H CPU. The software environment was configured with PyTorch 2.6.0, Python 3.10.0, CUDA 12.6, and cuDNN 9.5.1. The training parameters are presented in **Table 2**.

**Table 2:** Training parameters.

Parameter name	Parameter value
Batch Size	4
Initial Learning Rate	0.001
Optimizer	Adam
Epoch	200

#### 2.6.2 Model evaluation metrics

Quantitative evaluation of model performance is essential to verify the effectiveness of the algorithm in semantic and instance segmentation tasks of okra seedlings. Common evaluation metrics include Acc (Overall Accuracy), mIoU (mean Intersection over Union), and Rec (Recall), among which Acc and mIoU are adopted as the core indicators.

(1) Acc (Yu et al., 2022): Also known as PA (Point Accuracy). It represents the proportion of points with predicted categories consistent with the ground truth labels in all point clouds. The formula is defined in **Equation 13** as:

(13) $$\text{PA}+\frac{\text{TP}+\text{TN}}{\text{TP}+\text{TN}+\text{FP}+\text{FN}}$$

The numerator *TP*+*TN* denotes the total number of correctly classified points, and the denominator represents the total number of points in the point cloud.

TP (True Positive): Points correctly predicted as the positive category (e.g., okra seedlings).

TN (True Negative): Points correctly predicted as the negative category (e.g., background).

FP (False Positive): Negative points incorrectly classified as the positive category.

FN (False Negative): Positive points incorrectly classified as the negative category.

(2) mIoU (Ma et al., 2026): The mean Intersection over Union is calculated by averaging the IoU (Intersection over Union) of each category, which can comprehensively evaluate the segmentation performance across all categories and serves as the core metric for semantic segmentation. The IoU formula for a single category is given in **Equation 14** as:

(14) $${\text{IoU}}_{\text{i}}+\frac{{\text{TP}}_{\text{i}}}{{\text{TP}}_{\text{i}}+{\text{FP}}_{\text{i}}+{\text{FN}}_{\text{i}}}$$

where:

TP_i_: Number of points correctly predicted as the i-th category.

FP_i_: Points of other categories mistakenly predicted as the i-th category.

FN_i_: Points belonging to the i-th category but misclassified into other categories.

By averaging the IoU values of k categories, the mIoU is obtained as formulated in **Equation 15**:

(15) $$\text{mIoU}=\frac{1}{\text{k}}\sum_{\text{i}=0}^{\text{k}-1}{\text{IoU}}_{\text{i}}$$

## 3 Results

### 3.1 Sensitivity analysis and verification of DBSCAN parameters

To verify the rationality of DBSCAN parameter selection, a sensitivity analysis was conducted using the relative measurement errors of plant height, canopy width, and stem diameter as evaluation indicators. The results are presented in **Tables 3**, **4**. In **Table 3**, a moderate and reasonable value of minPts=10 was initially fixed, and the value of eps was adjusted accordingly. The results show that the measurement errors of all phenotypic indicators reach the minimum when eps=3. To further validate the rationality of the parameter combination, minPts was adjusted under the condition of eps=3, as listed in **Table 4**. It is found that the minimum measurement errors of all phenotypic indicators occur exactly at minPts=10, which demonstrates that the selected parameter combination possesses good rationality and robustness.

**Table 3:** Effect of eps on measurement error of phenotypic traits.

eps	Plant height relative error(%)	Canopy width relative error(%)	Stem diameter relative error(%)
1	0.45	0.40	0.82
2	0.28	0.25	0.53
3	0.21	0.19	0.42
4	0.32	0.29	0.70
5	0.63	0.58	1.05

**Table 4:** Effect of minPts on measurement error of phenotypic traits.

minPts	Plant height relative error(%)	Canopy width relative error(%)	Stem diameter relative error(%)
5	0.41	0.36	1.12
8	0.26	0.23	0.58
10	0.21	0.19	0.42
12	0.29	0.27	0.64
15	0.52	0.49	0.93

### 3.2 Comparative experiments of attention mechanisms

To verify the effectiveness of the introduced attention mechanism, comparative experiments were conducted on the PointNet++-MSG-C2F model by replacing SGE with several mainstream attention mechanisms, including SimAM (Wu et al., 2026), NAM (Cai et al., 2024), CBAM (Wisaeng, 2025), and SE (Huang Z. et al., 2025). The experimental results are summarized in **Table 5**. The results show that the model equipped with the SGE attention mechanism achieves higher accuracy and mIoU than other competing attention mechanisms, demonstrating superior segmentation performance.

**Table 5:** Comparative experiments of attention mechanisms.

Module name	Acc (%)	mIoU (%)
PointNet++-MSG-C2F+SGE	98.77	95.28
PointNet++-MSG-C2F+NAM	98.24	93.90
PointNet++-MSG-C2F+CBAM	97.99	92.97
PointNet++-MSG-C2F+SimAM	98.37	94.24
PointNet++-MSG-C2F+SE	98.46	94.66

### 3.3 Ablation experiment

Ablation experiments were carried out to verify the effectiveness of each improved module, and the results are shown in **Table 6**. It can be clearly observed that each incremental improvement of the proposed model enhances the algorithm performance to varying degrees.

**Table 6:** Ablation experimental results.

Method	Params(M)	FLOPs(G)	Mem(MB)	Time(ms)	Acc(%)	mIoU(%)
PointNet++-MSG	1.75	0.30	153.86	395.74	98.06	93.78
PointNet++-MSG+SGE	1.79	0.31	159.27	411.53	98.45	94.53
PointNet++-MSG+C2F	1.65	0.27	129.58	338.45	98.55	94.74
CSP-MSG Net	1.68	0.28	134.67	353.91	98.77	95.28

The baseline model PointNet++-MSG achieves relatively low Acc and mIoU. After embedding the SGE attention mechanism and replacing the MLP layer with the C2F module, the Acc reaches 98.45% and 98.55%, while the mIoU reaches 94.53% and 94.74%, respectively. Finally, when all optimization modules are integrated to construct the CSP-MSGNet, the Acc reaches 98.77%, an increase of 0.71 percentage points over the baseline, and the mIoU reaches 95.28%, with a promotion of 1.50 percentage points. Meanwhile, the parameter amount and computational complexity are significantly reduced compared with the baseline model. To maintain the optimal Acc and mIoU as well as sufficient feature representation capability, the proposed model retains appropriate feature capacity, which leads to a slight increase in computational complexity compared with the scheme using only the C2F module replacement. The above results fully validate the effectiveness and rationality of the combined application of each improved module.

Overall, the progressive improvement from PointNet++-MSG to CSP-MSGNet achieves continuous gains in Acc and mIoU and reflects the synergistic effect of each module in feature enhancement, model lightweighting and performance optimization. While maintaining high computational efficiency, the final model remarkably improves the detection and segmentation accuracy.

### 3.4 Comparison of semantic segmentation performance for okra seedlings using different models

**Figure 5:**
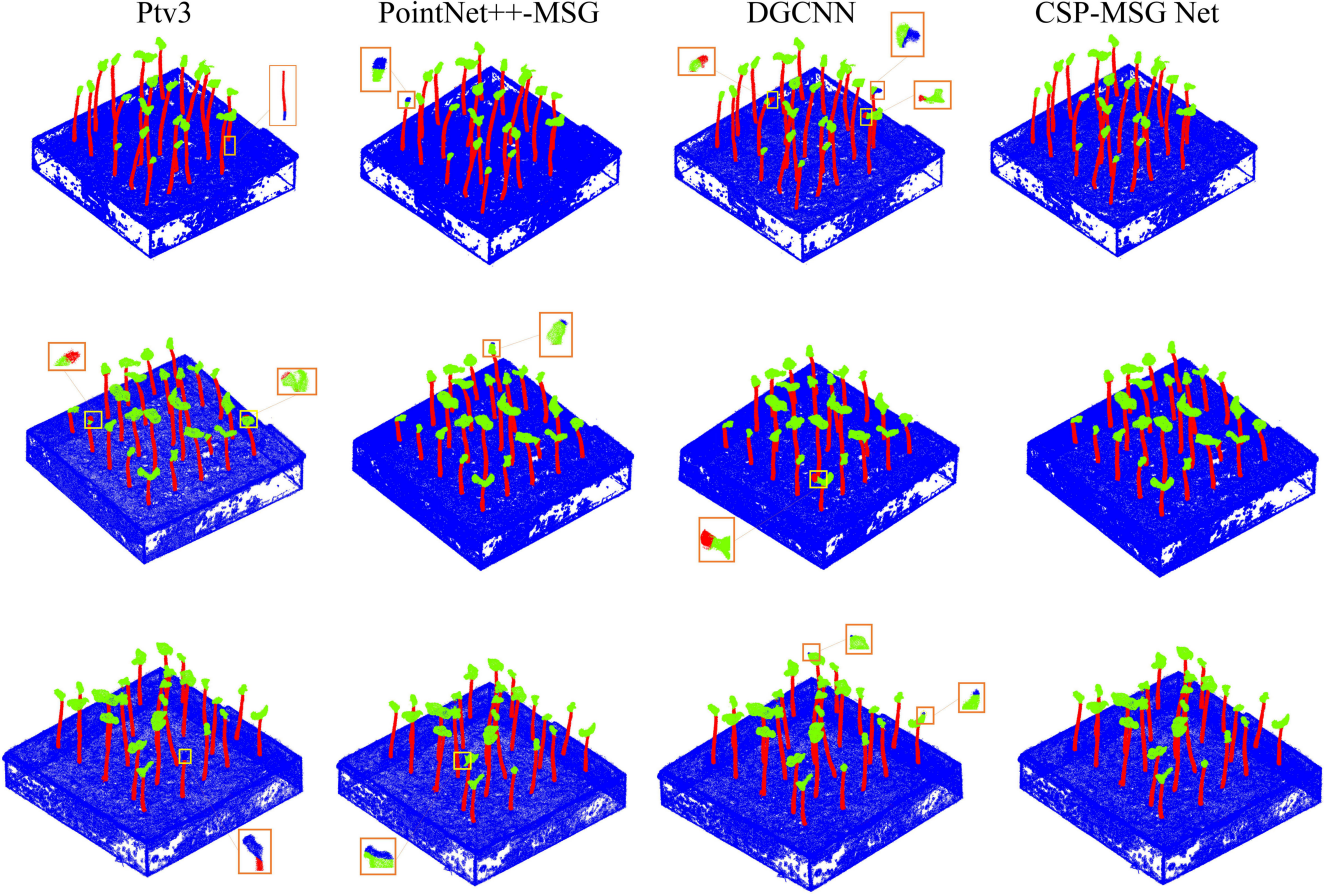
Visual comparison of semantic segmentation results of okra seedlings by different models.

To validate the effectiveness of the proposed CSP-MSG Net model, comparative experiments were conducted with several representative semantic segmentation models, including PTv3, PointNet++-MSG, and DGCNN. **Figure 5** presents the semantic segmentation visualization results of the four models for okra seedlings at different growth stages. It can be observed that PTv3, PointNet++ and DGCNN produce segmentation errors of varying degrees, whereas CSP-MSG Net achieves accurate and complete semantic segmentation, which demonstrates superior precision and reliability in this task.

**Table 7** quantitatively compares the proposed CSP-MSG Net with the three mainstream point cloud segmentation models in terms of segmentation performance and hardware overhead, with all experiments implemented under a unified hardware environment. The results indicate that increases in model parameters and computational complexity generally lead to higher memory consumption and inference latency. The proposed CSP-MSG Net outperforms PointNet++-MSG, DGCNN and PTv3 in terms of Params, FLOPs, memory usage and inference time, while achieving the best Acc and mIoU. These findings verify the effectiveness and superiority of the lightweight design of the proposed model.

**Table 7:** Parameter comparison of semantic segmentation performance for okra seedlings among different models.

Method	Params(M)	FLOPs(G)	Mem(MB)	Time(ms)	Acc(%)	mIoU(%)
PTv3	3.12	0.62	227.64	520.63	98.55	94.74
PointNet++-MSG	1.75	0.30	153.86	395.74	98.06	93.78
DGCNN	2.46	0.46	192.47	410.86	97.92	93.15
CSP-MSG Net	1.68	0.28	134.67	353.91	98.77	95.28

### 3.5 Validation of model segmentation performance and phenotypic parameter extraction method

#### 3.5.1 Evaluation of model segmentation performance

To verify the segmentation capability of the CSP-MSG Net model for different organs of okra seedlings, quantitative evaluation was carried out on three categories: pot, leaf, and stem, with the results listed in **Table 8**. The IoU of the proposed model for all three organs exceeds 93%, and the overall mIoU reaches 95.28%. The average values of Accuracy, Recall and F1-Score are 97.05%, 97.74% and 97.39%, respectively, indicating high segmentation accuracy and satisfactory robustness. Among them, the segmentation performance for pots and leaves is outstanding. Although the stem presents slightly lower indicators due to its slender morphology and susceptibility to occlusion, its accuracy still remains at a high level. The above results demonstrate that the proposed model can accurately distinguish and segment key organs of okra seedlings, laying a solid foundation for the extraction of phenotypic parameters such as plant height, canopy width and stem diameter, and ensuring the accuracy and reliability of subsequent phenotypic analysis.

**Table 8:** Evaluation metrics of segmentation performance for each organ of okra seedlings.

Part	IoU(%)	Acc(%)	Recall(%)	F1-Score(%)
Pot	96.91	98.52	99.03	98.77
Leaf	95.66	97.36	98.05	97.70
Stem	93.27	95.28	96.14	95.70
Mean	95.28	97.05	97.74	97.39

#### 3.5.2 Validation of phenotypic parameter extraction method

**Figure 6:**
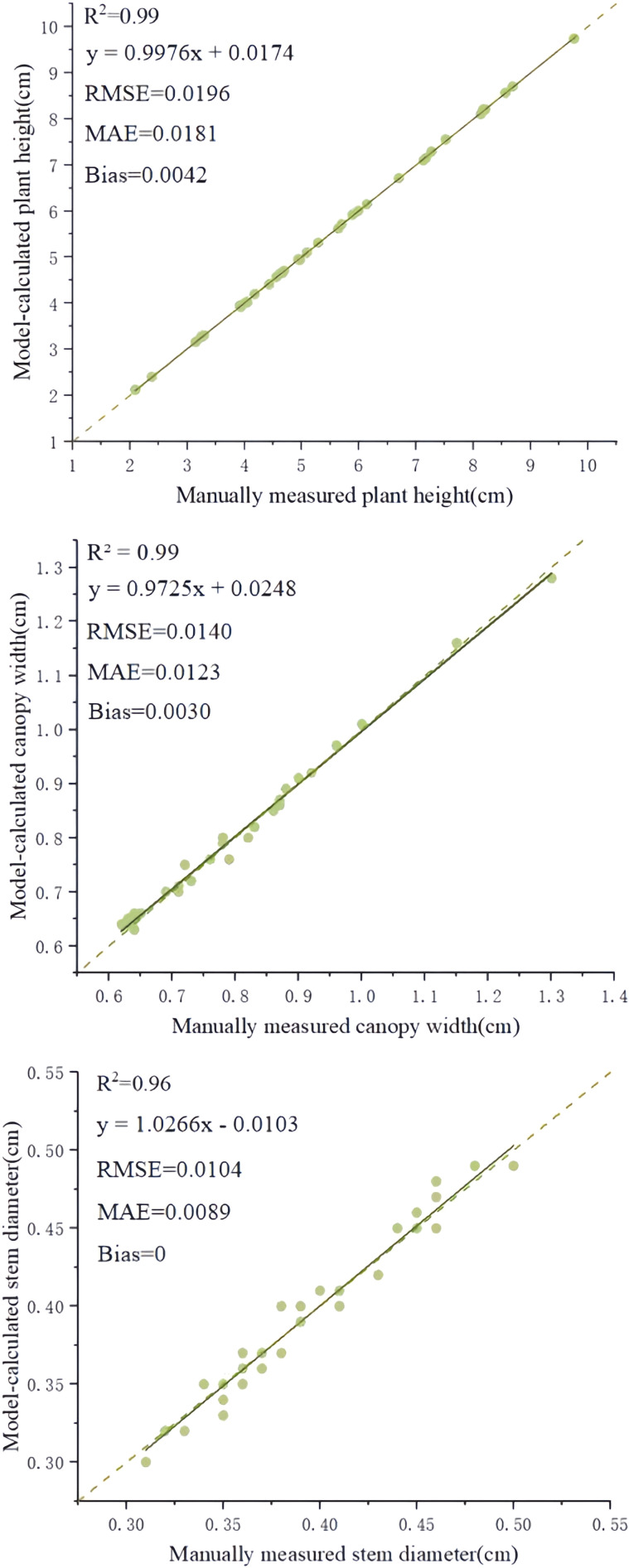
Algorithm effectiveness evaluation.

The coefficient of determination *R*^2^ (Goyal et al., 2026) is adopted to quantify the linear correlation between algorithm-calculated values and manual measured values. To further verify the accuracy of the okra seedling phenotypic parameter extraction method based on the CSP-MSG Net model, linear regression consistency analysis was performed between automatically extracted phenotypic data (plant height, canopy width, stem diameter) and manual measurements. The *y* = *x* ideal reference line was introduced for comparative evaluation, and RMSE, MAE, Bias and regression equation parameters were comprehensively used to assess the method performance. **Figure 6** presents the regression analysis results of the three phenotypic parameters in sequence. The *R*^2^ values of plant height, canopy width and stem diameter reach 0.99, 0.99 and 0.96, respectively. All regression slopes are close to 1 and intercepts are close to 0. Meanwhile, the RMSE, MAE and Bias of each parameter remain at extremely low levels with stable error distribution. The above results indicate that the phenotypic parameters extracted by the proposed method are in high consistency with manual measurements, possessing favorable extraction accuracy and reliability, which can meet the requirements of subsequent salt stress response analysis.

### 3.6 Experimental design

#### 3.6.1 Experimental procedure

**Figure 7:**
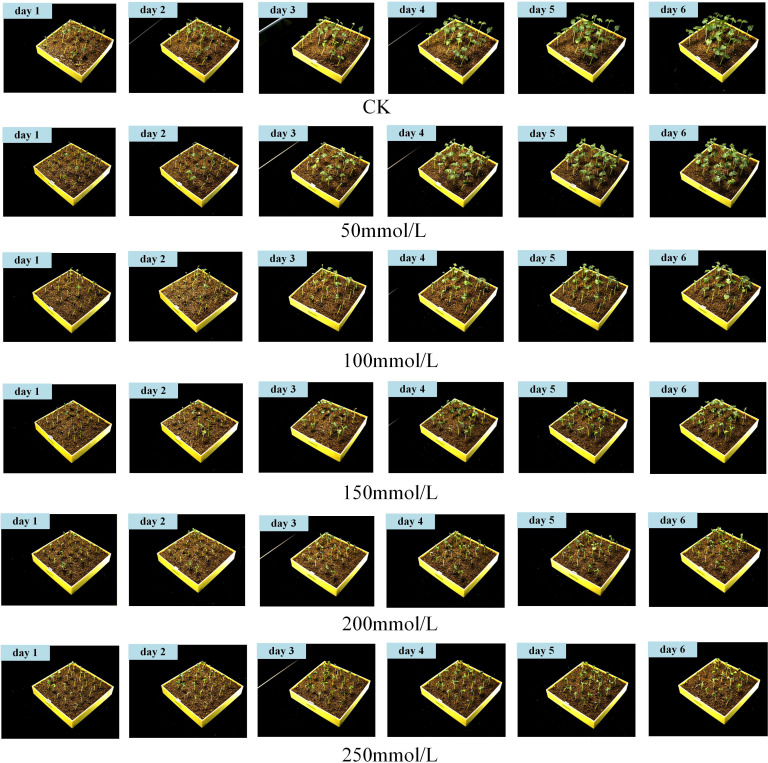
Axonometric images of okra seedlings growth.

**Figure 8:**
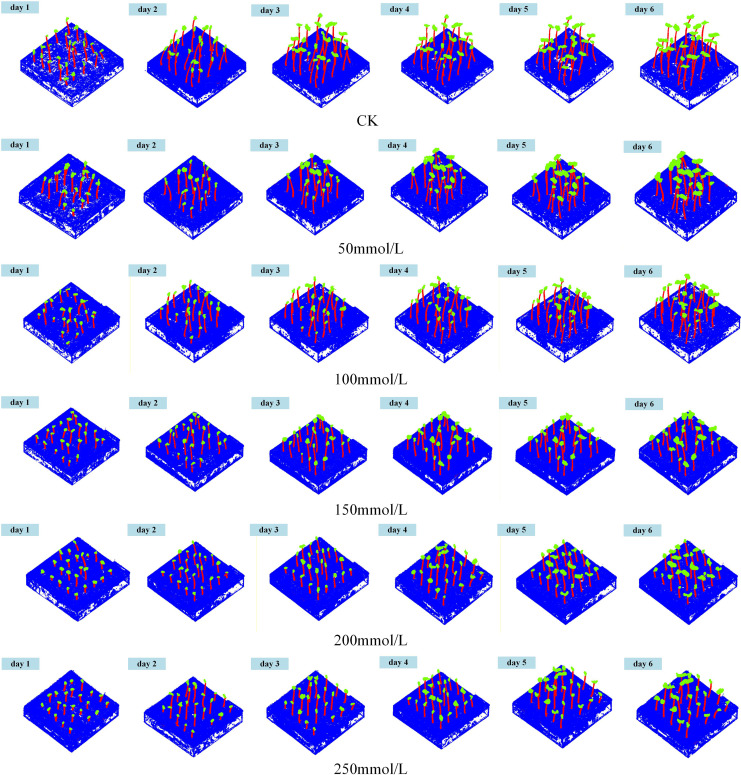
Semantic segmentation results using the CSP-MSG net model.

Based on the experimental platform, combined with the improved CSP-MSG Net model and phenotypic extraction technology, experimental research on the germination and growth of okra seedlings under salt stress was carried out. The dynamic changes of emergence rate, stem diameter, canopy width and plant height of okra seedlings within six days after sowing were observed and analyzed. Six concentration gradients of NaCl solution (0, 50 mmol/L, 100 mmol/L, 150 mmol/L, 200 mmol/L and 250 mmol/L) were set to simulate salt stress environments. The experiment was repeated three times. In each trial, six seedling trays corresponded to the six salt concentrations, with 25 seeds sown in each tray at a 5×5 planting density, resulting in 150 seeds per trial. A total of 450 okra seeds were used across three replicates to ensure statistical validity of the data. Due to the relatively slow emergence of okra seedlings, seedling emergence began on the sixth day after sowing. To investigate the phenotypic characteristics at the seedling stage, data collection started on the 6th day after sowing and was conducted every other day for six consecutive sampling days, ending on the 12th day after sowing. The oblique-view 3D images of okra seedling growth at six sampling days are shown in **Figure 7**, and the semantic segmentation results obtained by the CSP-MSG Net model are presented in **Figure 8**.

#### 3.6.2 Effects of different salt stress levels on phenotypic traits of okra seedlings

High salinity increases soil osmotic pressure, which restricts water absorption of seedlings and induces physiological drought, thereby inhibiting the growth and development of okra seedlings. To further explore the dynamic variations of phenotypic traits under different NaCl concentrations, three key indicators including canopy width, stem diameter and plant height were systematically measured and compared among six treatment levels: 0, 50 mmol/L, 100 mmol/L, 150 mmol/L, 200 mmol/L and 250 mmol/L. The comparative analysis of these indicators revealed the effects of salt stress on okra seedling growth, providing methodological support and data references for future multi-cultivar salt-stress phenotyping studies.

##### 3.6.2.1 Effect of different salt stress concentrations on plant height of okra seedlings

**Figure 9:**
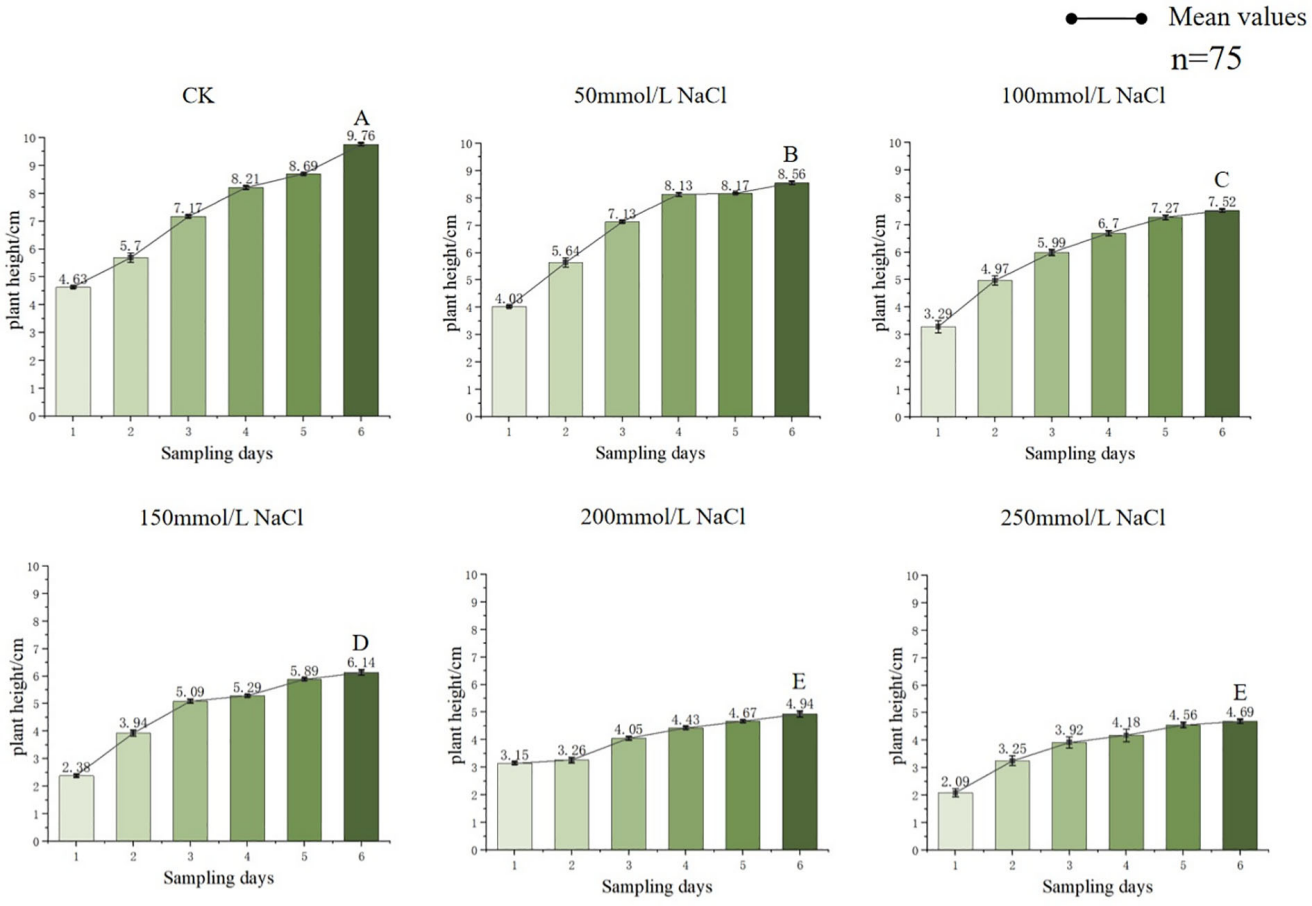
Temporal variation of average plant height under different salt stress concentrations. Uppercase letters A–E denote Tukey’s test results. Different letters indicate significant differences in plant height among treatments (P<0.05), same letters mean no significant difference (P>0.05).

**Figure 9** illustrates the dynamic changes in average plant height of okra seedlings with sampling days under different NaCl concentrations. The average plant height of all treatment groups increased continuously with the extension of sampling time, indicating that okra seedlings could still maintain growth under salt stress (Jose et al., 2025), while the growth rate gradually declined as salt concentration increased. The trend lines represent the sample average plant height of each salt treatment group, which integrates discrete data from three repeated experiments into a comparable benchmark. The lines intuitively reflect a dose-dependent inhibitory effect: the higher the salt concentration, the lower the average plant height. Meanwhile, the averaged trend eliminates random growth errors and improves data reliability, providing solid evidence for verifying the significant differences in plant height under various salt treatments. Taking the 6th sampling day as an example, the average plant height under each treatment was 9.76 cm (CK), 8.56 cm (50 mmol/L), 7.52 cm (100 mmol/L), 6.14 cm (150 mmol/L), 4.94 cm (200 mmol/L), and 4.69 cm (250 mmol/L). The results show that the control group always maintained the maximum plant height, and the inhibitory effect became more pronounced with the increase of salt concentration. The average plant height under 50 mmol/L was close to that of the control, suggesting that okra seedlings possess certain tolerance to low-concentration salt stress.

##### 3.6.2.2 Effect of different salt stress concentrations on canopy width of okra seedlings

**Figure 10:**
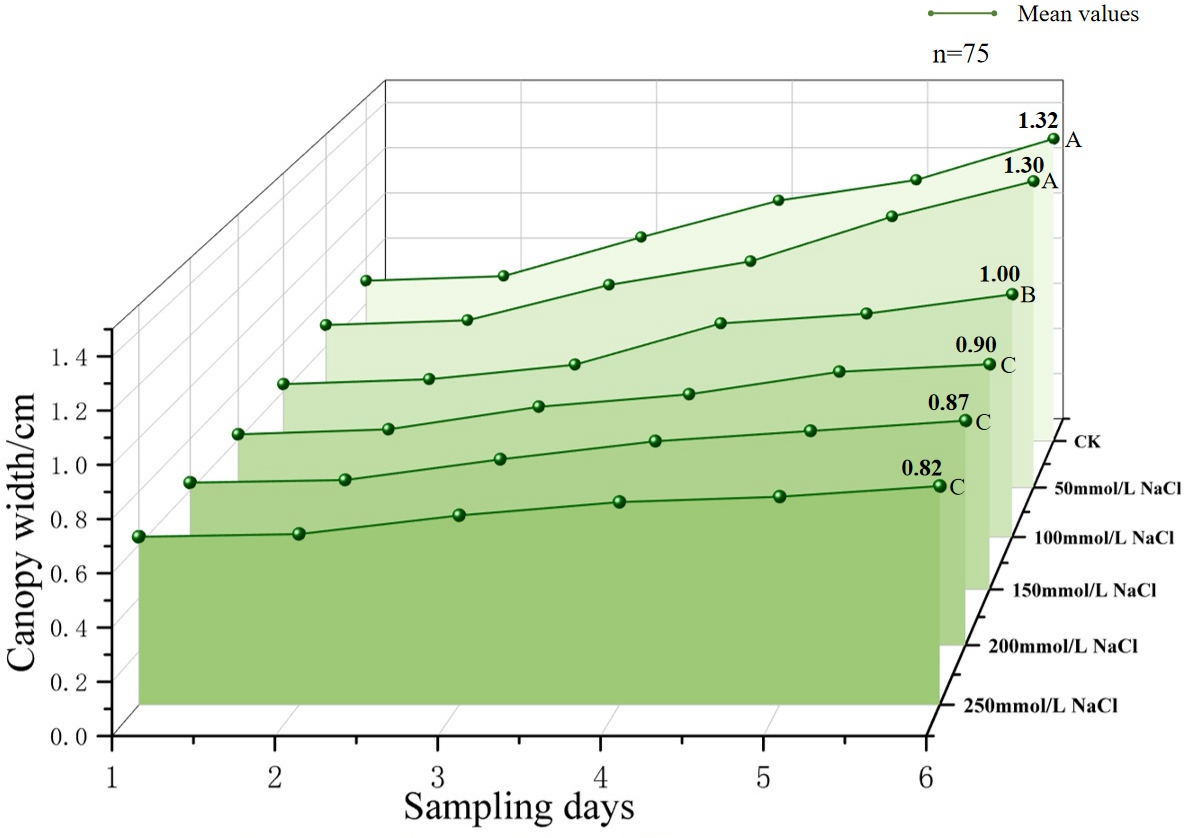
Temporal variation of average canopy width under different salt stress concentrations. Uppercase letters A–C denote Tukey’s test results. Different letters indicate significant differences in canopy width among treatments (P<0.05), same letters mean no significant difference (P>0.05).

It can be intuitively seen from **Figure 10** that the canopy width of okra seedlings under various salt stress concentrations shows a continuous increasing trend with the extension of sampling days after sowing, indicating that the canopy of okra seedlings can still grow normally and maintain a regular developmental process under salt stress. Comparing the canopy width between the control group (CK) and other salt treatment groups, the CK always maintains a significantly larger canopy width with the fastest growth rate. As the salt concentration rises, the canopy width decreases sequentially, presenting an obvious trend that the growth of canopy is increasingly inhibited with the increase of salt stress level. Taking the sixth sampling day as an example, the average canopy width under each treatment was 1.32 cm (CK), 1.30 cm (50 mmol/L), 1.00 cm (100 mmol/L), 0.90 cm (150 mmol/L), 0.87 cm (200 mmol/L), and 0.82 cm (250 mmol/L). At the early observation stage, the difference in canopy width among treatments was relatively small. As growth time prolonged, the gap gradually widened between the control group and salt-stressed groups, as well as between low-concentration and high-concentration treatments, demonstrating that the inhibitory effect of salt stress on canopy growth exhibits a time cumulative effect. The 250 mmol/L treatment group maintained the lowest canopy width throughout the experimental period with the flattest growth curve, indicating that salt stress at this concentration markedly restricted the canopy expansion rate and greatly suppressed the growth potential of okra seedlings.

##### 3.6.2.3 Effect of different salt stress concentrations on stem diameter of okra seedlings

**Figure 11:**
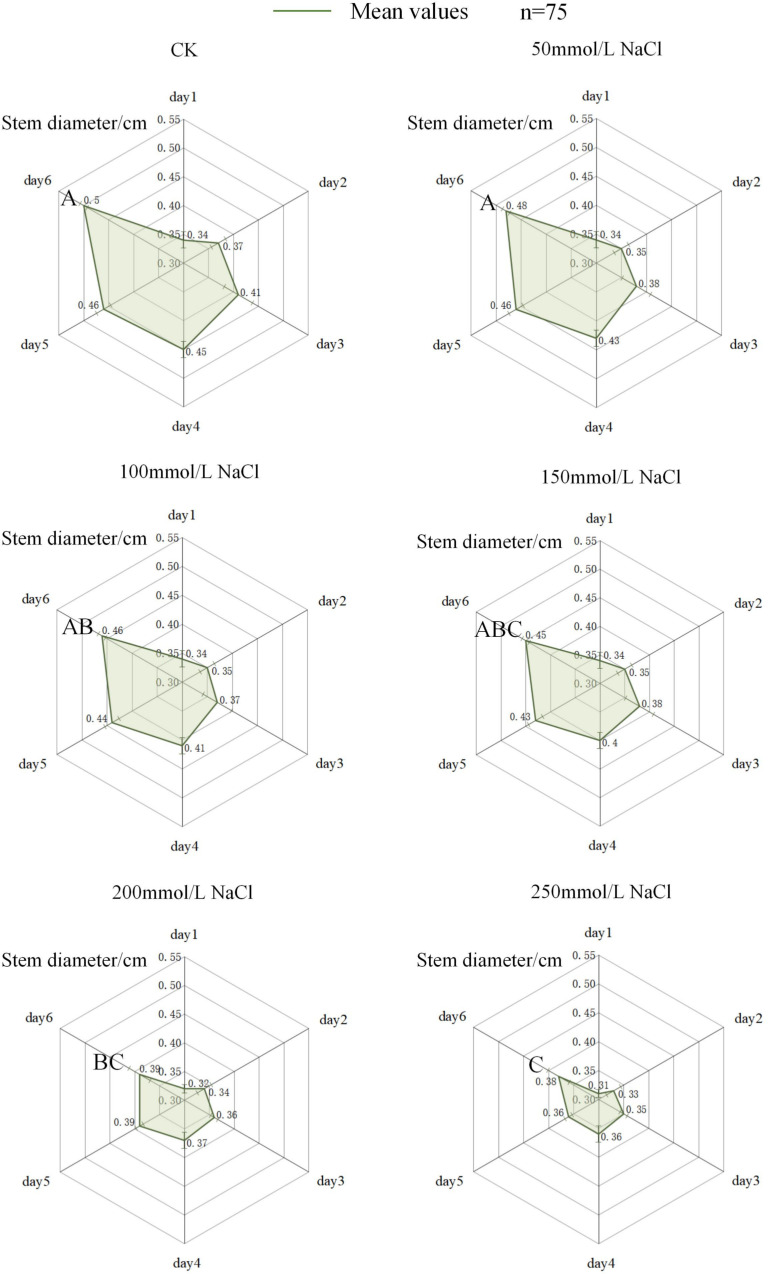
Temporal variation of average stem diameter under different salt stress concentrations. Uppercase letters A–C denote Tukey’s test results. Different letters indicate significant differences in stem diameter among treatments (P<0.05), same letters mean no significant difference (P>0.05).

**Figure 11** shows the dynamic changes in the average stem diameter of okra seedlings with sampling days under different NaCl concentrations. The average stem diameter of all treatment groups increased continuously with the advance of sampling time, indicating that okra seedlings could still undergo stem thickening under salt stress, while the growth rate gradually decreased as the salt concentration increased. The trend lines represent the mean stem diameter of okra seedlings in each salt treatment group, which integrates discrete data from three repeated experiments into a unified comparative benchmark. The curves intuitively present that the higher the salt concentration, the more the average stem diameter curve shrinks inward. Meanwhile, averaging repeated samples eliminates random growth errors and improves data reliability, providing an important basis for objectively judging the actual difference in stem diameter under various salt stress levels.

Taking the sixth sampling day as an example, the average stem diameter under each treatment was 0.50 cm (CK), 0.48 cm (50 mmol/L), 0.46 cm (100 mmol/L), 0.45 cm (150 mmol/L), 0.39 cm (200 mmol/L) and 0.38 cm (250 mmol/L). The results reveal that the control group always maintained the largest stem diameter, and the inhibitory effect became more prominent with the increase of salt concentration. The average stem diameter under 50 mmol/L was almost identical to that of the control group, demonstrating that okra seedlings have certain tolerance to low-concentration salt stress. As the salt concentration further rises, the inhibition on stem thickening is significantly intensified.

### 3.7 Analysis of variance and multiple comparisons on the effects of different salt concentrations on morphological indicators of okra seedlings

To further clarify the statistical differences in morphological indicators of okra seedlings among various salt concentration treatments, one-way analysis of variance (ANOVA) was performed on plant height, canopy width and stem diameter measured on the 6th day of salt stress. As shown in **Table 9**, different salt concentrations exerted extremely significant effects on plant height, canopy width and stem diameter of okra seedlings at day 6 (*p* < 0.001). The sum of squares among treatments for the three indicators was much larger than the within-group error sum of squares, indicating that the treatment effect induced by salt stress far exceeded the random experimental error. The differences among treatments were of high statistical significance.

**Table 9:** One-way analysis of variance for morphological indicators of okra seedlings under salt stress.

Index	Source of variation	Sum of squares (SS)	Degrees of freedom (df)	Mean square (MS)	F-value	P-value (Prob>F)
Plant height (cm)	Between	61.84024	5	12.36805	620.5	< 0.0001
	Within	0.2392	12	0.01993		
	Total	62.07944	17			
Canopy width (cm)	Between	0.73305	5	0.14661	162.9	< 0.0001
	Within	0.0108	12	0.00090		
	Total	0.74385	17			
Stem diameter (cm)	Between	0.03711	5	0.00742	10.3	< 0.001
	Within	0.00867	12	0.00072		
	Total	0.04578	17			

*p<*0.05 indicates significant difference; *p* < 0.001 indicates highly significant difference.

To further clarify the differential characteristics of morphological indicators of okra seedlings under different salt concentration treatments, Tukey’s multiple comparison test was conducted on the data of the 6th day, and the results were marked in **Figures 9**–**11**. Means with no common letters are significantly different at p<0.05. The results indicated that different morphological indicators exhibited distinct response patterns to salt stress.

## 4 Discussion

The results of this study showed that with increasing NaCl stress concentration, the growth rates of plant height, stem diameter and canopy width of okra seedlings decreased continuously. Low-concentration salt stress exerted slight inhibitory effects on seedling growth, whereas high-concentration salt stress significantly suppressed growth, clarifying the regulation rule of salt stress on phenotypic traits of okra seedlings. In this study, a lightweight phenotypic analysis system for okra seedlings under salt stress based on dual-view feature fusion was developed. Relying on three-dimensional point cloud technology, the system breaks the dimensional limitations of traditional two-dimensional detection. The proposed CSP-MSG Net achieves lightweight reconstruction through the C2F module and strengthens key feature extraction via the SGE attention mechanism. Combined with the DBSCAN clustering and the OBB bounding box algorithm, it enables accurate calculation of phenotypic parameters is achieved. This method effectively overcomes the shortcomings of traditional manual measurement, such as low efficiency, strong subjectivity, and low throughput, and provides methodological support for non-destructive and accurate phenotypic detection of okra seedlings under salt stress. Under the experimental conditions of this study, manual measurement and recording of plant height, stem diameter and canopy width for one pot of okra seedlings take an average of about 15 minutes; in contrast, the proposed model only consumes approximately 3 seconds to process a single pot sample, with the detection efficiency improved by nearly 300 times. It greatly reduces the labor and time costs of phenotypic detection. Nevertheless, there are still several limitations in this study:

1. Dataset construction relies heavily on three-dimensional point cloud reconstruction. During the late growth stage of okra seedlings, severe self-occlusion of leaves and mutual occlusion between adjacent plants remain challenging issues that hinder high-quality three-dimensional point cloud reconstruction.
2. Only three core phenotypic indicators (plant height, stem diameter, and canopy width) were selected to characterize the growth performance of okra seedlings under salt stress. Although the selected indicators show good validity and accuracy, the phenotypic detection index system remains relatively limited. In future research, more morphological and physiological indicators such as leaf area, biomass and plant structure can be supplemented to improve the non-destructive phenotypic detection system and enhance the comprehensiveness and applicability of the method.
3. The experimental material was limited to a single okra variety, Lingyun No.2. Inevitably, salt tolerance varies among different okra cultivars, and the present study only focuses on the germination and seedling stages. In the future, the proposed method can be further optimized and extended to the growth research of other okra cultivars and the whole growth period of okra.
4. The DBSCAN clustering parameters were optimized based on the dataset of this study, and its generalization performance on other datasets requires further verification. More robust point cloud denoising algorithms and adaptive hyperparameter optimization strategies can be introduced in subsequent research to further improve the stability and universality of the model.

## 5 Conclusions

Aiming at the low efficiency and poor accuracy of traditional manual measurement for okra phenotypic parameters, as well as the limitations of existing PointNet++ models including limited feature extraction capability, excessive parameters and poor robustness to complex point clouds, this study developed a high-throughput, accurate, and non-destructive method for 3D phenotypic analysis of okra seedlings under salt stress. Accordingly, this paper proposes a 3D point cloud processing and phenotypic parameter calculation pipeline for okra seedlings based on CSP-MSG Net:

1. An integrated experimental platform consisting of a full-time crop growth monitoring system and a three-view imaging system was applied. This platform supports automatic environmental control and continuous dual-view 3D data acquisition of okra from seed cultivation to seedling establishment. It not only provides a controllable and stable growth environment but also guarantees the integrity and validity of the collected 3D point cloud data.
2. A standardized 3D point cloud dataset of okra seedlings was constructed, and standardized preprocessing and point cloud processing workflows were formulated. Multiple data augmentation strategies were adopted to enrich dataset diversity, providing high-quality sample support for model training.
3. A lightweight point cloud segmentation architecture, CSP-MSG Net, was established based on PointNet++-MSG. The original MLP layers were replaced with C2F modules, and the SGE attention mechanism was incorporated. Combined with the DBSCAN clustering algorithm, organ-level semantic segmentation and individual plant instance segmentation of okra seedlings were realized. The network enhances local feature capture and channel weighting ability, thereby improving the accuracy and efficiency of point cloud segmentation.
4. A precise calculation method for core phenotypic parameters of okra seedlings was established. The Oriented Bounding Box (OBB) was introduced to realize point cloud scale restoration and high-precision calculation of phenotypic parameters. Comparative analysis between manual measurements and model calculations verifies the reliability of the proposed method, which provides a solid technical support for non-destructive quantitative detection of morphological traits of okra seedlings under salt stress.
5. We conducted a growth experiment on okra seedlings under multi-concentration NaCl stress, with six salt concentration gradients and three biological replicates. Using the CSP-MSG Net model, we analyzed the dynamic growth patterns of plant height, stem diameter, and canopy width of okra seedlings under different salt stress levels and confirmed the inhibitory effect of salt stress on the growth rate of okra seedlings. This study provides methodological references for non-destructive and accurate phenotyping of salt stress in multi-cultivar okra in future research.

In conclusion, the proposed method can achieve efficient and accurate 3D phenotypic analysis of okra seedlings and serve as a reliable technical approach for non-destructive quantitative detection of okra phenotypes under salt stress. Meanwhile, this study still has certain limitations: leaf occlusion interferes with point cloud reconstruction; only a single okra variety and seedling stage were investigated; and the phenotypic detection indicators are relatively limited. In future work, the point cloud anti-occlusion capability will be optimized, the research will be extended to multiple varieties and the whole growth period, more phenotypic indicators will be supplemented, and the method will be further applied to phenotypic analysis of more crop species.

## Data Availability

The raw data supporting the conclusions of this article will be made available by the authors, without undue reservation.
